# CO_2_ and temperature decoupling at the million-year scale during the Cretaceous Greenhouse

**DOI:** 10.1038/s41598-017-08234-0

**Published:** 2017-08-23

**Authors:** Abel Barral, Bernard Gomez, François Fourel, Véronique Daviero-Gomez, Christophe Lécuyer

**Affiliations:** 1 Laboratoire de Géologie de Lyon, CNRS UMR 5276, Université Lyon 1, Villeurbanne, 69622 France; 2 Institut Universitaire de France, Paris, France

## Abstract

CO_2_ is considered the main greenhouse gas involved in the current global warming and the primary driver of temperature throughout Earth’s history. However, the soundness of this relationship across time scales and during different climate states of the Earth remains uncertain. Here we explore how CO_2_ and temperature are related in the framework of a Greenhouse climate state of the Earth. We reconstruct the long-term evolution of atmospheric CO_2_ concentration (*p*CO_2_) throughout the Cretaceous from the carbon isotope compositions of the fossil conifer *Frenelopsis*. We show that *p*CO_2_ was in the range of ca. 150–650 ppm during the Barremian–Santonian interval, far less than what is usually considered for the mid Cretaceous. Comparison with available temperature records suggest that although CO_2_ may have been a main driver of temperature and primary production at kyr or smaller scales, it was a long-term consequence of the climate-biological system, being decoupled or even showing inverse trends with temperature, at Myr scales. Our analysis indicates that the relationship between CO_2_ and temperature is time scale-dependent at least during Greenhouse climate states of the Earth and that primary productivity is a key factor to consider in both past and future analyses of the climate system.

## Introduction

Greenhouse gas emissions resulting from human activity are considered to be an important driver of the current global warming. Accurate understanding of this cause-effect relationship is essential to propose realistic scenarios of the future evolution of Earth’s climate, but the human monitoring history of these variables may be very tenuous to properly take into account the relative importance of their large time-scale dynamics into the climate system. Geological records offer an opportunity to address this time-scale issue as they provide track of the long-term dynamics of atmospheric greenhouse gas contents and surface temperature throughout Earth’s history. An analysis of this relationship throughout the whole Phanerozoic pointed out atmospheric *p*CO_2_ as the main multi-scale driver of global warming^1^. However, recent improvement and refining of methods to reconstruct *p*CO_2_ evidence that this conclusion was based on outdated imprecise estimates^2, 3^. Additionally, some factors involved in long-term climate dynamics such as O_2_ have been put forward to challenge this paradigm^4^. This raises questions about the extent to which we are correctly interpreting the relative influence of atmospheric *p*CO_2_ forcing on climate^5^.

The analysis of the relationship between *p*CO_2_ and temperature for the Phanerozoic as a whole may not be pertinent because it comprised shifts between drastically different climate states of the Earth in which the relative impact and the dynamics of atmospheric CO_2_ may have strongly differed. Of these, Greenhouse is considered to be the default planetary climate state of the Earth, prevailing in more than 70% of the Phanerozoic^6^. The Cretaceous is one of the longest and most studied Greenhouse periods of Earth’s history, with an extensive background of *p*CO_2_^7–10^ and temperature^11–13^ reconstructions. This makes the Cretaceous an exceptional ‘laboratory’ of the past to explore the relationship between the two factors. Nevertheless, *p*CO_2_ reconstructions made so far for the Cretaceous defined temporal trends that are not consistent with those described for temperature^11–13^ or the carbon cycle dynamics evidenced by carbon isotopes^14–16^. This raises questions about how accurate are *p*CO_2_ reconstructions made so far and how significant was the relative contribution of *p*CO_2_ to temperature evolution during the Cretaceous.

Carbon isotopes are relevant tools for describing past carbon cycle and CO_2_ dynamics^14–20^. Carbon isotope compositions of terrestrial plants are particularly interesting as they are strongly linked to atmospheric CO_2_, which is the main source of carbon assimilated by plants via photosynthesis. The recent construction of models relating carbon isotope fractionation by plants and *p*CO_2_^19–21^ coupled with the good preservation of the pristine carbon isotope signal within plant fossil materials^22–25^, make carbon isotope compositions of plant fossils valuable proxies to analyze the evolution of atmospheric *p*CO_2_ during past periods.

Herein the long-term evolution of atmospheric *p*CO_2_ during the Cretaceous is reconstructed based on the carbon isotope compositions of leaves of the fossil conifer *Frenelopsis* (δ^13^C_leaf_). *p*CO_2_ is estimated from twelve stages spanning the Barremian–Santonian interval (129.4–83.6 Ma) during which major disturbance events of the carbon cycle related to CO_2_ release to the atmosphere^14, 15, 18^ and major variations in temperature^11–13^ described for the Cretaceous took place. We explore the correspondence between estimated *p*CO_2_ trends and those previously published for temperature to analyze the relative influence of *p*CO_2_ forcing on climate during the Cretaceous.

## Results and Discussion

### Carbon isotope compositions of the fossil conifer *Frenelopsis*

**Figure 1:**
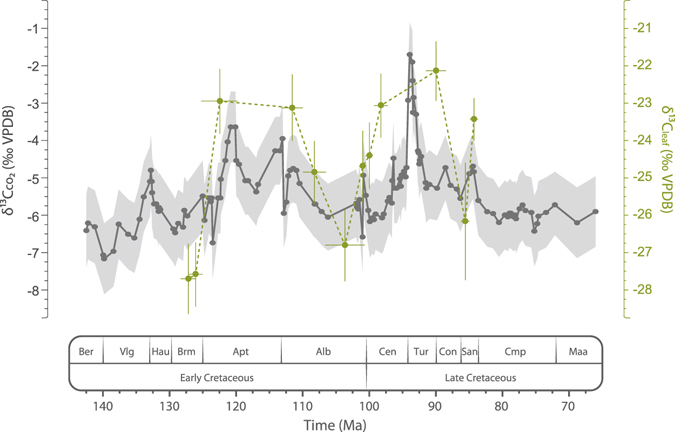
Comparison of the carbon isotope compositions of leaves of the fossil conifer *Frenelopsis* (δ^13^C_leaf_) obtained for each studied episode with the evolution of the carbon isotope composition of atmospheric CO_2_ (δ^13^C_CO2_) throughout the Cretaceous estimated by Barral *et al*.^16^. Envelopes and bar errors correspond to ±1σ.

δ^13^C_leaf_ values from the twelve studied stages range from −27.7 to −22.1‰ in average (Fig. 1; Table 1). In most of these stages, δ^13^C_leaf_ values show similar standard deviations (ca. 0.9‰), thus reflecting a similar range of local environmental variability. Santonian stages are the only exceptions, which may reflect higher climatic instability related to the development of Oceanic Anoxic Event (OAE) 3. Carbon isotope compositions of *Frenelopsis* in the Barremian, upper Albian and lower Santonian stages are notably ^13^C-depleted compared to others, even though it is not significant in the latter case (Fig. 1). These lower δ^13^C_leaf_ values are consistent with decreases in the carbon isotope composition of atmospheric CO_2_ (δ^13^C_CO2_) recently described by Barral *et al*.^16^ (Fig. 1). Overall, there is a good agreement between δ^13^C_leaf_ and δ^13^C_CO2_ curves, though trends in the former are generally magnified. This magnification is more pronounced during the Cenomanian-Turonian interval, coinciding with a critical increase in global temperatures^11^, which may have slightly reduced carbon isotope fractionation by plants (Δ^13^C_leaf_) by reducing the discrimination effect of RuBisCO against ^13^C^26, 27^.

**Table 1:** δ^13^C_leaf_, δ^13^C_CO2_, Δ^13^C_leaf_ and *p*CO_2_ values for the twelve studied Cretaceous stages. μ and σ correspond to mean and standard deviation, respectively.

**Locality**	Age	δ^13^C_leaf_ (‰)			δ^13^C_CO2_ (‰)		Δ^13^C_leaf_ (‰)		*p*CO_2_ (ppm)	
		μ	σ	n	μ	σ	μ	σ	μ	σ
Hautrage	upper lower Barremian–lower upper Barremian	−27.7	0.9	30	−6.1	0.5	22.3	1.1	476.5	135.2
Uña	upper Barremian	−27.6	0.9	30	−5.7	0.7	22.5	1.1	502.2	155.1
Mas de la Parreta	uppermost Barremian–lower Aptian	−23.0	0.9	30	−5.8	0.7	17.6	1.1	191.6	37.6
El Soplao	lower Albian	−23.1	0.9	30	−5.0	0.3	18.6	1.0	225.6	39.4
San Just	lower–middle Albian	−24.9	0.8	30	−5.8	0.5	19.6	1.0	272.3	53.8
Reillo	upper Albian	−26.8	1.0	30	−5.8	0.3	21.6	1.1	408.9	99.0
Archingeay (A1sl–A)	uppermost Albian	−24.7	1.0	26	−5.7	0.4	19.5	1.1	265.8	53.3
Archingeay (A2sm1–2)	lowermost Cenomanian	−24.4	0.9	30	−5.9	0.4	18.9	1.0	240.9	43.9
La Buzinie	uppermost lower–lowermost middle Cenomanian	−23.1	0.9	30	−5.9	0.5	17.6	1.0	190.5	34.5
Infiesto	upper Turonian–Coniacian	−22.1	0.8	30	−5.1	0.4	17.4	0.9	185.0	30.1
Piolenc	lower Santonian	−26.2	1.6	17	−5.2	0.6	21.5	1.7	432.5	220.3
Belcodème	upper Santonian	−23.4	0.6	30	−4.8	0.5	19.1	0.8	246.0	34.5

### Atmospheric CO_2_ concentration during the Cretaceous

**Figure 2:**
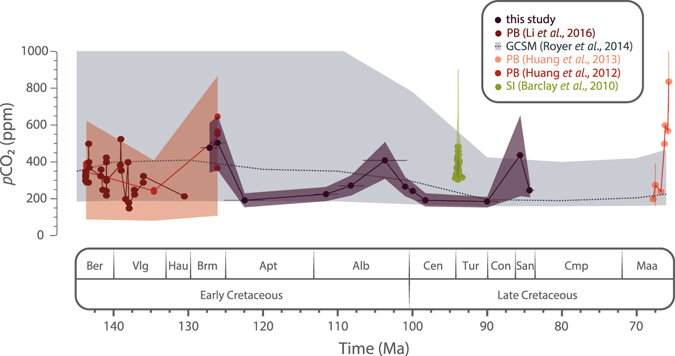
Comparison of the δ^13^C_leaf_-based *p*CO_2_ estimates with most recent proxy-based and model-based *p*CO_2_ reconstructions for the Cretaceous^3, 7–10^. Envelopes and bar errors represent ±1σ. GCSM: GEOCARBSULF Model reconstructions; PB: Paleosol Barometer reconstructions; SI: Stomatal Index reconstructions.

Δ^13^C_leaf_ values inferred from δ^13^C_leaf_ and concomitant δ^13^C_CO2_ values (Table 1) were used to estimate *p*CO_2_ values. *p*CO_2_ estimates are in the range of ca. 150–650 ppm for the Barremian–Santonian interval, and the resolution is better than 55 ppm (i.e. 1σ, standard deviation) for most of the stages (Fig. 2; Table 1). This range of values is consistent with the conclusions by Franks *et al*.^20^ from a method based on universal equations of leaf gas exchange using stomatal anatomy and carbon isotope ratios of fossil leaves suggesting *p*CO_2_ values below 1000 ppm during most of the Phanerozoic, although their conclusions remain controversial^28–30^. Our *p*CO_2_ estimates average ca. 300 ppm for the studied interval, which is in accordance with the most recent revision of GEOCARBSULF model^3^ and the range of values obtained in most recent works dealing with proxy-based reconstructions^7–10^ (Fig. 2). All these reconstructions suggest that atmospheric CO_2_ concentrations were comparable to those of present-day atmosphere, which is a conclusion in deep contrast with the classical view that the Cretaceous period was characterized by atmospheric CO_2_ concentrations considerably higher than today^31^. This is also consistent with several other approaches. Boron isotope compositions (δ^11^B) of marine biogenic carbonates are closely related to *p*CO_2_ and pH^32–34^. Higher atmospheric concentrations would imply more CO_2_ dissolution into the ocean, causing a lowering of seawater pH and δ^11^B of marine biogenic carbonates^33, 34^. However, δ^11^B of Cretaceous and present-day brachiopods as well as ophiolitic serpentinites are close to each other^35^, thus suggesting that seawater pH and atmospheric *p*CO_2_ were of similar magnitude. Analysis of reef abundance and diversity throughout the Phanerozoic also agrees with our results, indicating that during the Cretaceous no significant long-term rise in ocean acidification, reflecting a long-term rise in *p*CO_2_, took place^36^. Only discrete short-term events of ocean acidification have been described during the Cretaceous based on marine calcifiers, associated with major Cretaceous OAEs and Hothouse episodes^37, 38^.

### Was *p*CO_2_ a long-term driver of temperature during the Cretaceous?

**Figure 3:**
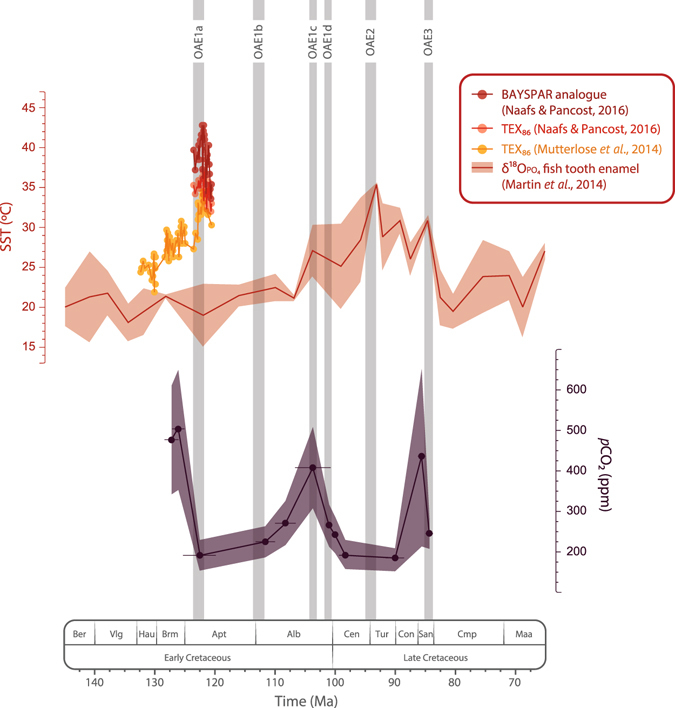
Reconstructed *p*CO_2_ compared with available sea surface temperature (SST) records^11–13^ for the Cretaceous. Vertical grey bars represent major Oceanic Anoxic Events (OAE) occurring during the Cretaceous^18^. Envelopes represent ±1σ.

Several events of volcanic CO_2_ release into the atmosphere linked to the emplacement of large igneous provinces have been inferred from pronounced negative shifts of both carbon and strontium isotope compositions of marine carbonates^14, 38^ associated with the main Cretaceous OAEs^14, 18, 37^. These CO_2_ release events are also evident regarding the evolution of δ^13^C_CO2_ throughout the Cretaceous^16^ (Fig. 1) and are most likely responsible for temperature increases at the kyr scale^12, 13^. The biggest of these CO_2_ release events in terms of the magnitude of carbon isotope excursions was related to OAE1b and lasted ca. 25 kyr^14, 39^. This event was responsible for a fast warming of the atmosphere of ca. 0.3 °C and followed by cooler climatic conditions during a period of ca. 45 kyr^39^. Greater kyr-scale increases in temperature of ca. 6–7 °C have been described associated with the CO_2_ release events related to OAE1a^12, 13^ and OAE2^11^ (Fig. 3). However, our results indicate that drastic Myr-scale *p*CO_2_ drawdowns took place during the warmest time intervals described for the Cretaceous^11–13^: a drawdown of ca. 310 ppm (from ca. 500 to ca. 190 ppm) during the upper Barremian-lower Aptian interval (ca. 7.5 Myr), and ca. 225 ppm (from ca. 410 ppm to ca. 190 ppm) during the upper Albian-lower Cenomanian interval (ca. 5 Myr) (Fig. 3). In fact, at the Myr-scale, trends in *p*CO_2_ are even inverse compared to temperature during significant time intervals such as the upper Barremian–lower Aptian (considering the higher resolution temperature curves based on TEX_86_ proxy^12, 13^) and the upper Albian–Santonian in which *p*CO_2_ minima are coeval with thermal maxima (Fig. 3). If *p*CO_2_ was rather low and usually decoupled from temperature at the Myr-scale, it may not have been the main long-term driver of global warming during the Cretaceous.

### Time scale dependency of the relationship between *p*CO_2_ and temperature

Episodes of enhanced primary production have been associated with the kyr-scale CO_2_ release events that took place during major OAEs and the warmest intervals of the Cretaceous^40–44^. Barclay *et al*.^7^ showed that a 20% increase in *p*CO_2_ during OAE2 triggered extensive primary productivity leading to a global increase in rates of organic carbon burial, which eventually resulted in a *p*CO_2_ decrease down to 26% in ca. 100 kyr. This indicates that, as a consequence of a critical CO_2_ release event to the atmosphere, primary productivity can account for *p*CO_2_ drawdowns of at least up to the 10^5^ yr scale. The time resolution of our record is not high enough to fully discuss *p*CO_2_ trends on the kyr scale; however, when the time uncertainty of our estimates allows clear perusal, *p*CO_2_ levels are consistently low well after the events of CO_2_ release to the atmosphere described in the literature^18, 45^ (Fig. 3). This indicates that the aforementioned primary productivity effect may exceed the kyr scales resulting in a negative balance or a low-level stasis of *p*CO_2_ up to the Myr scales. If primary production were responsible for a long-term decrease in atmospheric *p*CO_2_ during the Cretaceous, we should find a co-varying increase in atmospheric *p*O_2_ as a product of long-term enhanced photosynthesis. This is in accordance with the long-term trends obtained by the recent revision of the GEOCARBSULF model, which describes both a drawdown of *p*CO_2_ and an increase in *p*O_2_ levels from the earliest stages of the Cretaceous up to the mid Cretaceous^3^. Poulsen *et al*.^4^ emphasized the importance of O_2_ in long-term climate dynamics as under low *p*O_2_ conditions shortwave scattering by clouds and air molecules is reduced; this implies a significant increase in surface shortwave forcing, leading to increase of atmospheric water vapour and consequent enhanced greenhouse forcing which increases global surface temperatures. Thus, their simulations predict that temperature increases under low *p*O_2_ and high *p*CO_2_ conditions. However, during the Cretaceous temperature was maintained high during the late Albian–Santonian interval coinciding with relatively high *p*O_2_^3^ and low *p*CO_2_ conditions^3, 7^ (Fig. 3). This observation reflects that both *p*O_2_ and *p*CO_2_ parameters may not be enough to explain long-term evolution of temperature.

In addition to the conclusions of previous work on atmospheric CO_2_ dynamics at kyr time scales^7^ our results indicate that the relationship between CO_2_ and temperature was probably scale-dependent during the Cretaceous. In the framework of a Greenhouse climate state functioning, CO_2_ appears to have been a main driver of global warming and primary production at timescales up to the kyr^7^. However, our results indicate that it was decoupled from temperature and most likely a long-term consequence of the climate-biological system at Myr scales. Direct extrapolation of these conclusions to time intervals corresponding to different climate states of the Earth is not pertinent because C cycle dynamics, and thus the evolution of *p*CO_2_, might be strongly modulated by different global scale functioning of climate factors. For instance, temperature strongly affects carbon sequestration by living organisms^26, 27^, and global oceanic and atmospheric circulation patterns are involved in heat transport^46^ and distribution of climate zones^47^ that strongly influence primary productivity. Different dynamics and relative effects of those factors within different climate states of the Earth^6^ may result in different functioning of atmospheric CO_2_ dynamics. However, nowadays anthropogenic CO_2_ release into the atmosphere also constitutes a main driver of climate change at a human time scale. At larger scales, primary productivity appears to have been an important driver of atmospheric CO_2_ concentration during both interglacial^48^ and glacial intervals^49^. Further work on the Myr-scale interrelationships between primary productivity, CO_2_ and temperature within these different climate state intervals of the Earth is needed to explore how universal are the conclusions obtained herein. Primary production constitutes a major factor involved in climate dynamics and that regulates the balance between *p*O_2_ and *p*CO_2_. Thus, productivity must be taken into consideration in the evaluation of the relative effect of O_2_ and CO_2_ on climate dynamics. Present-days anthropogenic CO_2_ release event can be compared in magnitude with those of the Cretaceous, reflected in a ca. −1.8‰ shift in δ^13^C_CO2_ since the industrial revolution^50^ against the ca. −2‰ shift associated with OAE1b^16^ (Fig. 1). However, its critically shorter time of development makes it a more intense disturbance event, and so its forthcoming consequences are difficult to predict. Evaluating the response time of primary production to cushion CO_2_ forcing will be critical to allow the prediction of climate evolution and its impact in the near future of life on Earth.

## Methods

Twelve stages corresponding to eleven sites from the Cretaceous of western Europe were used for *p*CO_2_ reconstruction (see Supplementary Fig. S1). The sites represent similar terrestrial environments in which the fossil conifer genus *Frenelopsis* was present, and they are distributed over a narrow palaeolatitude band (30–40° N) of the same climate zone throughout the time range analyzed^51^ (see Supplementary Table S1). These stages and sites were selected to reduce as much as possible environmental biases on δ^13^C_leaf_.

*Frenelopsis* was chosen as a *p*CO_2_ proxy because it was frequent and abundant in Cretaceous ecosystems^52–55^. *Frenelopsis* is a valuable material for performing stable carbon isotope analysis due to its resistance to decay and diagenesis. It provides accurate δ^13^C_leaf_ values, which are comparable to those of living plant leaves^25, 56, 57^.

*Frenelopsis* leaves were treated with HCl 32% following the protocol recommended by Barral *et al*.^25^. Thirty *Frenelopsis* leaves per locality were separately reduced to powder using an agate mortar. Three replicates of ca. 100 μg per leaf were randomly selected and weighted using a precision balance Sartorius ME36S and loaded into tin capsules. Carbon isotope analyses were performed using a continuous flow IRMS configuration, being He the carrier gas, using a EuroEA3028™–HT elemental analyzer working in combustion mode and interfaced with an IP60 isotope ratio mass spectrometer. Carbon isotope ratios were calibrated against the international standard IAEA-C4 (δ^13^C = −23.96‰) and casein (δ^13^C = −22.67‰) as an internal reference. Based on repeated analysis of standards, analytical reproducibility was better than ±0.2‰.

*p*CO_2_ values were estimated for each stage using the equation relating *p*CO_2_ to δ^13^C_leaf_ described by Schubert and Jahren^19, 21^ (r = 0.94):

$${{\rm{\Delta }}}^{13}{{\rm{C}}}_{{\rm{leaf}}}=[(28.26)(0.22)(p{{\rm{CO}}}_{2}+23.9)]/[28.26+(0.22)(p{{\rm{CO}}}_{2}+23.9)]$$

Δ^13^C_leaf_ values were estimated from δ^13^C_leaf_ and concomitant δ^13^C_CO2_ values by using the equation proposed by Farquhar *et al*.^58^:

$${{\rm{\Delta }}}^{13}{{\rm{C}}}_{{\rm{leaf}}}={{\rm{\delta }}}^{13}{{\rm{C}}}_{{\rm{Co2}}}-{{\rm{\delta }}}^{13}{{\rm{C}}}_{{\rm{leaf}}}/1+({{\rm{\delta }}}^{13}{{\rm{C}}}_{{\rm{co2}}}/1000)$$

δ^13^C_CO2_ values were taken from the δ^13^C_carb_-based δ^13^C_CO2_ estimates provided by Barral *et al*.^16^. We avoid discrimination bias due to different physiological functioning related to taxonomic specificity by using always the same taxon, and thus δ^13^C_leaf_ faithfully represents environmental variability. In order to avoid misrepresented *p*CO_2_ estimates due to subtle uncorrelations between the beds from which δ^13^C_CO2_ and δ^13^C_leaf_ were obtained, we accounted for δ^13^C_CO2_ variability in the uncertainty timespan of the *Frenelopsis* localities by averaging and estimating cumulated error for each stage. This was made by randomly drawing 10,000-repetition Gaussian distributions for each single case within the interval of uncertainty of a given stage, defined by the pre-determined means and standard deviations of each case, and calculating the mean and the standard deviations of the 10,000 randomly generated sets. This procedure was also implemented to include the associated errors to each δ^13^C_leaf_, δ^13^C_CO2_ and Δ^13^C_leaf_ values into the aforementioned equations to account for all sources of error in the resolution of *p*CO_2_ estimates.

## Electronic supplementary material

Supplementary Information
