# Supplementary material for: CO2 and temperature decoupling at the million-year scale during the Cretaceous Greenhouse
Source: Sci Rep. 2017 Aug 23;7:8310. doi: 10.1038/s41598-017-08234-0 (PMC5569102; doi:10.1038/s41598-017-08234-0)
Supplement: Supplementary file 1 — Supplementary Information [file 41598_2017_8234_MOESM1_ESM.pdf]

Supplementary information materials for

CO_2 and temperature decoupling at the million-year scale during the Cretaceous Greenhouse

Abel Barral, Bernard Gomez, François Fourel, Véronique Daviero-Gomez and Christophe Lécuyer

Figure S1. Geographical map of the eleven localities corresponding to the twelve Cretaceous stages from which *Frenelopsis* samples were studied (original maps from d-maps.com; Europe map: ttp://d-maps.com/carte.php?num_car=30075&lang=es; Belgian regions: http://d-maps.com/carte.php?num_car=17839&lang=es; French regions: http://d-maps.com/carte.php?num_car=18001&lang=es; Spanish regions: http://d-maps.com/carte.php?num_car=18017&lang=es).

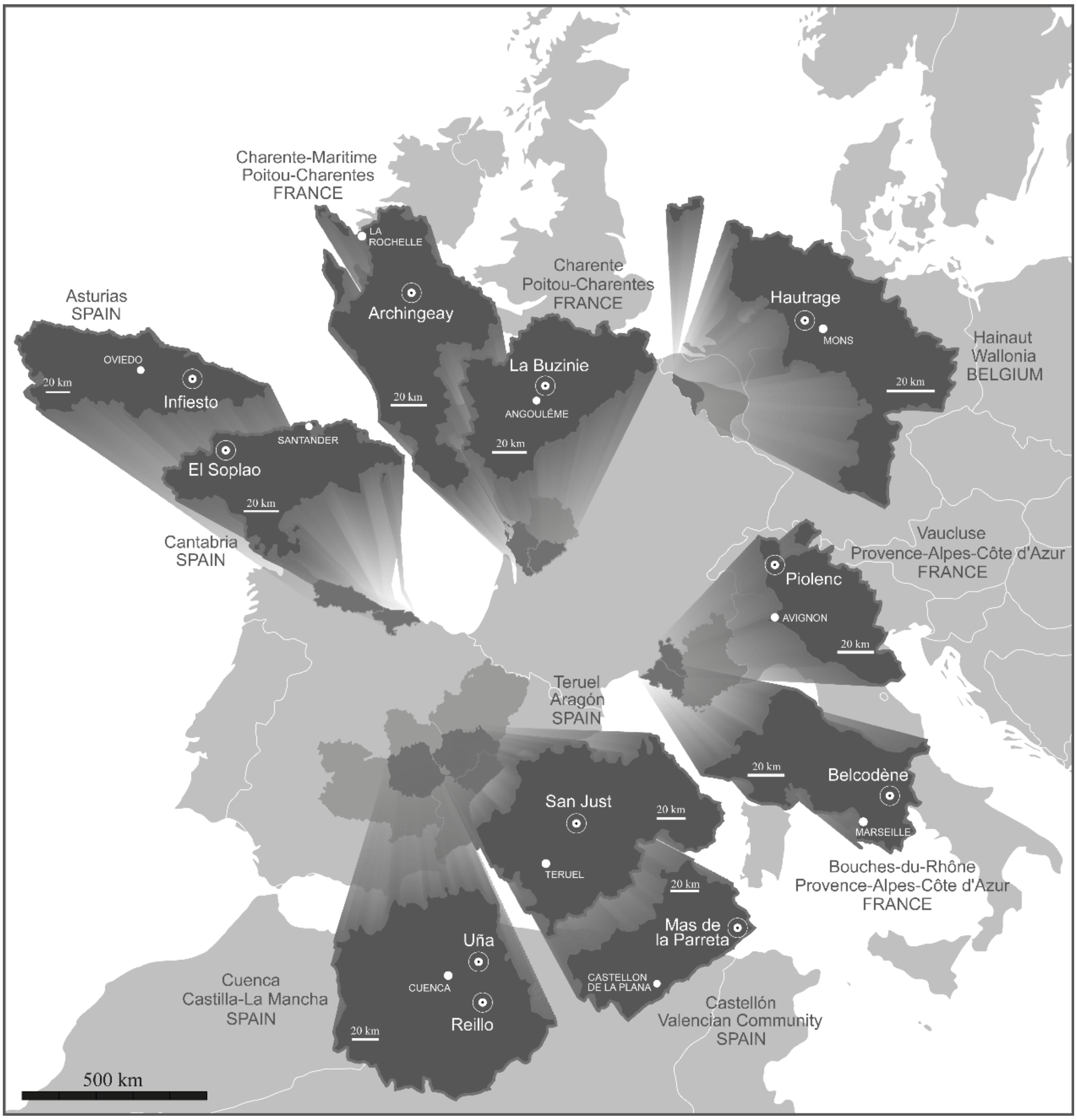

Table S1. Geographical, geological and palaeoenvironmental settings of the localities from which *Frenelopsis* samples were studied.

Locality	Area	Country	Stratigraphy	Age	Bed	Lithology	Paleoenvironment	Refs.
Hautrage	Hainaut	Belgium	Hautrage Clays Fm.	upper lower Barremian-lower upper Barremian	HTpt–4	Sideritic dark clays	Floodplain with very poor development of soil	1-6
Uña	Cuenca	Spain	La Huérguina Fm.	upper Barremian	3	Coaly laminated clays draping massive sandy layers	Flooding event in a cycle of deltaic progradation-retrogradation in the marginal zone of a lake	7
Mas de la Parreta	Castellón	Spain	Arcillas de Morella Fm.	uppermost Barremian-lower Albian	CMP-3	Red clays	Freshwater swamp or mangrove	8,9
El Soplao	Cantabria	Spain	Las Peñosas Fm.	lower Albian	FA3	Silty clays	Delta plain	10,11
San Just	Teruel	Spain	Escucha Fm.	lower-middle Albian	SJpt–1	Grey-black clays	Freshwater swamp plain subenvironment within an upper deltaic plain	12-15
Reillo	Cuenca	Spain	Arenas de Utrillas Fm.	upper Albian	-	Red clays	Floodplain	16
Archingeay	Charente-Maritime	France	Charente-Maritime deposits	uppermost Albian	A1sl-A	Clays intersected by fine sandy beds	Brackish or marine water influenced environment, mangrove	17
				lowermost Cenomanian	A2sm1–2	Blue-Grey laminated clays	Freshwater or brackish water influenced environment, mangrove	17,18
La Buzinie	Charente	France	-	uppermost lower-lowermost middle Cenomanian	B3/C	Lignitic clays	Paralic environment	19
Infiesto	Asturias	Spain	Infiesto Fm.	upper Turonian-Coniacian	-	-	-	20
Piolenc	Vaucluse	France	-	lower Santonian	C	Sandy clays	Supratidal ponds or marshes	21
Belcodène	Bouches-du-Rhône	France	-	upper Santonian	-	Laminated marls	Coastal environment	22

Supplementary Information References

1. Quinif, Y., Méon, H. & Yans, J. Nature and dating of karstic filling in the Hainaut Province (Belgium). Karstic, geodynamic and paleogeographic implications. *Geodin. Acta* 19, 73–85 (2006).
2. Thiry, M. *et al.* Continental France and Belgium during the early Cretaceous: paleoweatherings and paleolandforms. *Bull. Soc. Géol. Fr.* 177, 155–175 (2006).
3. Spagna, P., Dupuis, C. & Yans, J. Sedimentology of the Wealden clays in the Hautrage quarry. *Mem. Geol. Surv. Belg.* 55, 35–44 (2008).
4. Spagna, P., Yans, J. & Schnyder, J. in *Bernissart Dinosaurs and Early Terrestrial Ecosystems* (ed. Godefroit, P.) 464 (Series: Life of the Past. Indiana University Press, Bloomington, USA., 2012).
5. Yans, J. *et al.* Carbon-isotope of fossil wood and dispersed organic matter from the terrestrial Wealden facies of Hautrage (Mons Basin, Belgium). *Palaeogeogr. Palaeoclimatol. Palaeoecol.* 291, 85–105 (2010).
6. Barral, A. *et al.* Local-scale analysis of plant community from the Early Cretaceous riparian ecosystem of Hautrage, Belgium. *Palaeogeogr. Palaeoclimatol. Palaeoecol.* 443, 107–122 (2016).
7. Gomez, B., Martín-Closas, C., Méon, H., Thévenard, F. & Barale, G. Plant taphonomy and palaeoecology in the lacustrine Uña delta (Late Barremian, Iberian Ranges, Spain). *Palaeogeogr. Palaeoclimatol. Palaeoecol.* 170, 133–148 (2001).
8. Gámez, D., Paciotti, P., Colombo Piñol, F., Salas, R. & others. La Formación Arcillas de Morella (Aptiense inferior), Cadena Ibérica oriental (España): caracterización sedimentológica. (2003).
9. Villanueva-Amadoz, U., Santisteban, C. & Santos-Cubedo, A. Age determination of the Arcillas de Morella Formation (Maestrazgo Basin, Spain). *Hist. Biol.* 27, 389–397 (2014).
10. Najarro, M., Peñalver, E., Rosales, I., Daviero-Gomez, V. & others. Unusual concentration of Early Albian arthropod-bearing amber in the Basque-Cantabrian Basin (El Soplao, Cantabria, Northern Spain): palaeoenvironmental and palaeobiological implications. *Geol. Acta Int. Earth Sci. J.* 7, 363–387 (2009).
11. Najarro, M. *et al.* Review of the El Soplao amber outcrop, Early Cretaceous of Cantabria, Spain. *Acta Geol. Sin.-Engl. Ed.* 84, 959–976 (2010).

12. Querol, X., Salas, R., Pardo, G. & Ardevol, L. Albian coal-bearing deposits of the Iberian Range in northeastern Spain. *Geol. Soc. Am. Spec. Pap.* 267, 193–208 (1992).
13. Peñalver, E., Delclòs, X. & Soriano, C. A new rich amber outcrop with palaeobiological inclusions in the Lower Cretaceous of Spain. *Cretac. Res.* 28, 791–802 (2007).
14. Villanueva-Amadoz, U., Pons, D., Diez, J. B., Ferrer, J. & Sender, L. M. Angiosperm pollen grains of San Just site (Escucha Formation) from the Albian of the Iberian Range (north-eastern Spain). *Rev. Palaeobot. Palynol.* 162, 362–381 (2010).
15. Barral, A., Lécuyer, C., Gomez, B., Fourel, F. & Daviero-Gomez, V. Effects of chemical preparation protocols on $\delta^{13}C$ values of plant fossil samples. *Palaeogeogr. Palaeoclimatol. Palaeoecol.* 438, 267–276 (2015).
16. Meléndez, M. N. El Cretácico de la región de Cañete-Rincón de Ademuz (provincias de Cuenca y Valencia). (Departamento de Estratigrafía y Geología Histórica, Facultad de Ciencias Geológicas, Universidad Complutense, 1983).
17. Gomez, B. *et al.* Floristic assemblage from the Albian-Cenomanian of Charente-Maritime (SW France). *Ann. Paléontol.* 90, 147–159 (2004).
18. Gomez, B., Coiffard, C., Dépré, É., Daviero-Gomez, V. & Néraudeau, D. Diversity and histology of a plant litter bed from the Cenomanian of Archingeay–Les Nouillers (southwestern France). *Comptes Rendus Palevol* 7, 135–144 (2008).
19. Batten, D. J., Colin, J.-P. & Néraudeau, D. Megaspores from mid Cretaceous deposits in western France and their biostratigraphic and palaeoenvironmental significance. *Rev. Palaeobot. Palynol.* 161, 151–167 (2010).
20. Bernárdez Rodríguez, E. Los dientes de seláceos del Cretácico de la Depresión Central Asturiana. (Departamento de Geología, Universidad de Oviedo, 2002).
21. Gomez, B., Barale, G., Saad, D. & Perrichot, V. Santonian angiosperm-dominated leaf-assemblage from Piolenc (Vaucluse, Sud-Est de la France). *Comptes Rendus Palevol* 2, 197–204 (2003).
22. Saint Martin, S., Saint Martin, J.-P., Girard, V. & Néraudeau, D. Organismes filamenteux de l'ambre du Santonien de Belcodène (Bouches-du-Rhône, France). *Annales de Paléontologie* 99, 339–360 (2013).
